# Dengue fever: a decade of burden in Iran

**DOI:** 10.3389/fpubh.2024.1484594

**Published:** 2024-10-23

**Authors:** Zahra Heydarifard, Fatemeh Heydarifard, Fatemeh Sadat Mousavi, Milad Zandi

**Affiliations:** ^1^Department of Virology, Faculty of Medicine, Lorestan University of Medical Sciences, Khorramabad, Iran; ^2^Department of Veterinary, Faculty of Veterinary Medicine, Lorestan University, Khorramabad, Iran; ^3^Department of Microbiology and Immunology, Faculty of Veterinary Medicine, University of Tehran, Tehran, Iran; ^4^Department of Microbiology, Faculty of Medicine, Guilan University of Medical Sciences, Rasht, Iran

**Keywords:** Dengue virus, Aedes mosquito, Iran, vector control, seroprevalence

## Abstract

Since the first reported traveler case of Dengue in Tehran in 2008, the virus has gradually spread across Iran, driven by factors including international travel, climate change, and Aedes mosquito vectors. The disease has manifested in various forms, ranging from mild fever to severe dengue, with notable cases identified in Sistan and Baluchistan Province. Despite the increasing prevalence, Iran faces significant challenges in surveillance, diagnosis, and vector control. This perspective comprehensively analyzes dengue virus epidemiology in Iran, emphasizing the need for enhanced public health strategies, including genomic surveillance, targeted interventions, and health education. The findings highlight the critical importance of addressing these challenges to mitigate the potential for large-scale dengue epidemics and protect public health.

## Introduction

Since the first traveler case of dengue was reported in Tehran in 2008, Dengue virus (DENV) has gradually spread throughout Iran, with a continuous increase in reported cases (1). As a mosquito-borne flavivirus transmitted by Aedes (Stegomyia) *aegypti* and Ae. (Stegomyia) *albopictus* mosquitoes, DENV shares its vectors with other notable viruses such as chikungunya (CHIKV), Zika (ZIKV), and yellow fever (2). DENV has four serotypes (DENV-1 to DENV-4), each with distinct genotypes. DENV-2, frequently associated with severe infections and epidemics, consists of six genotypes: Asian/American, Asian I, Asian II, Cosmopolitan, American, and sylvatic (3).

Dengue fever presents a wide range of clinical manifestations, from asymptomatic infections to mild flu-like syndromes, dengue fever (DF), and severe dengue disease (SDD), encompassing dengue hemorrhagic fever (DHF) and dengue shock syndrome (DSS) (4). Neurological complications may include encephalopathy, encephalitis, seizures, intracranial hemorrhage, and Guillain-Barré syndrome (GBS) (5). With a 2.5% mortality rate, dengue poses a significant global health concern, with the World Health Organization reporting an 8-fold increase in cases between 2000 and 2019 (from 505,430 to 5.2 million) (6). In 2023, a global resurgence was observed, characterized by a marked rise in outbreaks in previously unaffected regions. Climate change-induced mosquito population growth is expected to further expand dengue-endemic areas and increase the at-risk population (7). One dengue vaccine, Dengvaxia^®^, is approved in the United States for individuals aged 9–16 with a history of dengue infection living in high-risk areas (8, 9).

this viewpoint offers a comprehensive analysis of the past decade of dengue fever in Iran. By exploring epidemiological patterns, viral evolution, disease burden, and public health measures, we aim to contribute to the global understanding of DENV. These insights can inform policy and guide future research, ultimately strengthening efforts to prevent, control, and eliminate dengue fever in Iran and beyond.

## Epidemiological trajectories of Dengue virus in Iran

Dengue fever has become a significant public health concern in Iran since 2008, following the confirmation of the first case in a 58-year-old woman from Tehran with a travel history to Malaysia (1). Subsequent investigations revealed a gradual spread of the Dengue virus (DENV) across the country, raising questions about the epidemiological trajectories of this mosquito-borne flavivirus. A retrospective study conducted on 300 previously collected samples (2000–2012), primarily from the Sistan and Baluchistan province, detected 15 (5%) positive cases through serology and 3 (1%) positive cases through serology plus polymerase chain reaction (PCR) for DF. Notably, 7 (46.7%) of the 15 seropositive cases had no travel history outside Iran, with six originating from Sistan and Baluchestan, a region adjacent to Pakistan with a hot-dry climate conducive to Aedes mosquito emergence (10). Further evidence of DENV circulation emerged from Chabahar (2013), where 30 out of 540 samples tested positive, and 11 were equivocal for DENV IgG antibodies using the ELISA method (11). Additionally, a 32-year-old woman with a recent travel history to India was diagnosed with DENV in October 2015, highlighting the role of international travel in disease transmission (12). In Zahedan (2013–2015), a study involving 60 patients revealed evidence of past or recent DENV exposure in 13 individuals, with IgM antibodies detected in 6.5% of patients (13). Notably, none of these patients had a travel history outside Iran, emphasizing the potential for local DENV transmission (13). A case report from 2016 documented a 39-year-old woman with a travel history to Malaysia being diagnosed with DENV in Sabzevar city (14). Further evidence of DENV circulation was found in a study conducted between December 2016 and November 2017 at the National Measles Laboratory in Tehran, where 82 out of 1,306 tested sera were dengue seropositive (15) (Table 1).

**Table 1:** Summary of published articles reporting DENV in Iran.

Authors/years	Type of study	Province	Samples	Serology detection	Molecular detection	Travel history to endemic country
Mardani/2008 (1)	Case report	Tehran	A 58-year-old woman suspected	Positive IgM	–	Malaysia
Chinikar/2012 (10)	Retrospective study	Mostly form Sistan and Baluchistan	300 suspected patients for DENV	10 (3.3%) IgM seropositive, 10 (3.3%) IgG seropositive	3 (1%) positive by RT-PCR	Mostly Malaysia, Thailand and India
Aghaei/2013 (11)	Cross-sectional	Sistan and Baluchistan	540 volunteer healthy donors	7.6% positive by ELISA and 5.9% positive by IFA	–	No one of positive samples had a travel history
Baniasadi/2015 (12)	Case report	Tehran	A 32-year-old woman suspected	Positive IgM	confirm by RT-PCR	India
Heydari/2015 (13)	Cross -sectional	Sistan and Baluchistan	60 suspected patients	4 (6.5%) IgM positive, 3 (5%) IgG positive and 3(5%) NS1 antigen positive	–	None of patients had travel history outside Iran
Ebrahimi/2016 (14)	Case report	Khorasan	A 39-year-old woman suspected	Positive IgM	–	Malaysia
Tavakoli/2017 (15)	Cross- sectional	All province	1,306 suspected case for measles and rubella	82 (6.2%) patients positive for IgM by ELISA	–	Not available

Recently, the Ministry of Health and Medical Education in Iran reported 137 dengue fever cases from 15 May to 10 July 2024. Only one patient succumbed to the infection, with most infected patients having a travel history to endemic countries such as Pakistan, the United Arab Emirates, Oman, and West Africa (Benin). Notably, twelve patients from Bandar Lengeh, Hormozgan Province, had no travel history to foreign countries, indicating the presence of mosquito vectors in Iran (16).

## The rise of Dengue virus endemic in Iran: Sistan and Baluchistan province at the forefront

**Figure 1:**
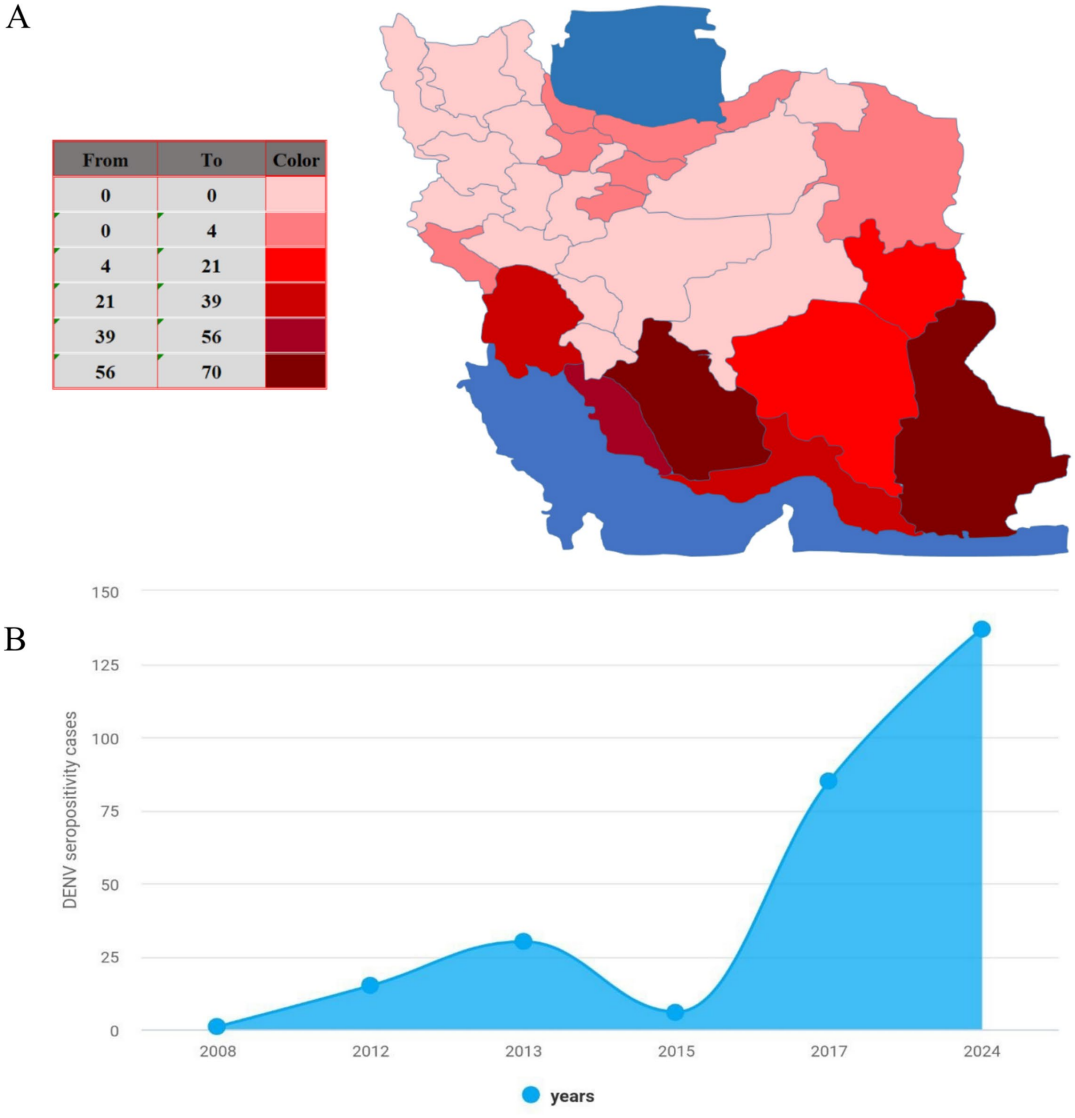
Epidemiology of dengue in Iran. (A) The map shows cumulative dengue cases (ELISA-confirmed) in Iran from 2008 to 2024. (B) Annual reported seropositive dengue cases (ELISA-confirmed) in Iran from 2008 to 2024.

Located in the southeastern region of Iran, Sistan and Baluchistan province has witnessed a surge in Dengue virus (DENV) endemic cases, positioning itself as a focal point in the country (13) (Figure 1). This development is a result of various factors working together, such as the province’s geographical position, the presence of Aedes mosquito vectors, the region’s climate, and its international trade connections.

One major contributing factor to the spread of DENV in Sistan and Baluchistan is its geographical proximity to Pakistan, a country also dealing with DF endemics. The shared border between Iran and Pakistan allows for the possible movement of infected individuals across the border, heightening the probability of DENV transmission in the province. A study showcased this connection, with 46.7% of 15 seropositive cases having no travel history outside Iran, and six cases originating from Sistan and Baluchestan province (10).

Another contributor to the potential for DENV transmission in Iran is the presence of Aedes mosquito vectors, particularly *Aedes albopictus* and *Aedes aegypti*. Ae. *albopictus* was first reported in Chabahar, Sistan and Baluchestan Province, while Ae. *aegypti* was observed in Bandar Lengeh, Hormozgan Province (17, 18). Although the establishment of these Aedes mosquito species in Iran remains unconfirmed, continued monitoring and surveillance are necessary due to the predicted likelihood of their presence in studied area based on modeling. It appears that these species have not yet adapted to the climatic conditions of these areas (19, 20).

Additionally, climate change projections indicate that northern and southern regions of Iran, including Sistan and Baluchistan, are more susceptible to DENV transmission due to favorable climatic conditions for mosquito vectors (21). Studies conducted in Iran have shown a higher prevalence of Ae. *albopictus* in the southern regions of Sistan and Baluchestan Province (17, 22). This highlights the importance of incorporating climate data into public health strategies to counteract the impact of climate change on disease dynamics.

International trade, particularly maritime transportation, also significantly influences the potential spread of DENV and its vectors. Shipping containers and vessels may serve as ideal habitats for Aedes mosquitoes, allowing them to disperse to new regions (23). With Sistan and Baluchistan strategically located along international trade routes, regular and accurate monitoring of entomological activity at these entry points is crucial to minimize the risk of DENV and its vectors being introduced and spread (24).

## Preliminary genomic surveillance of Dengue virus in Iran

In Iran, understanding the patterns and spread of Dengue virus (DENV) is essential for managing and controlling the disease. However, several factors pose challenges to obtaining an accurate picture of its prevalence, including limitations in healthcare infrastructure, diagnostic capabilities, and the co-existence of similar diseases. Over-reliance on passive surveillance may result in underestimating the true impact of DENV in Iran. Furthermore, the scarcity of trained laboratory professionals and entomologists hampers the ability to identify and track cases effectively. Unequal access to diagnosis can also lead to underreporting and misdiagnosis. The presence of other arboviral diseases with similar symptoms, such as Chikungunya virus (CHIKV), West Nile virus (WNV), and Crimean-Congo haemorrhagic fever (CCHF), complicates the diagnostic process for clinicians, contributing to an incomplete understanding of the burden of these co-existing diseases (25–27). To address these challenges, Iran must improve its diagnostic capabilities by implementing point-of-care testing and cost-effective multiplex reverse transcription polymerase chain reaction tests. Strengthening collaborations between local, national, and international health agencies, as well as research institutions, is vital for building capacity, reducing inequalities in healthcare infrastructure, and gaining a more comprehensive understanding of DENV and other arboviral diseases (28, 29).

**Figure 2:**
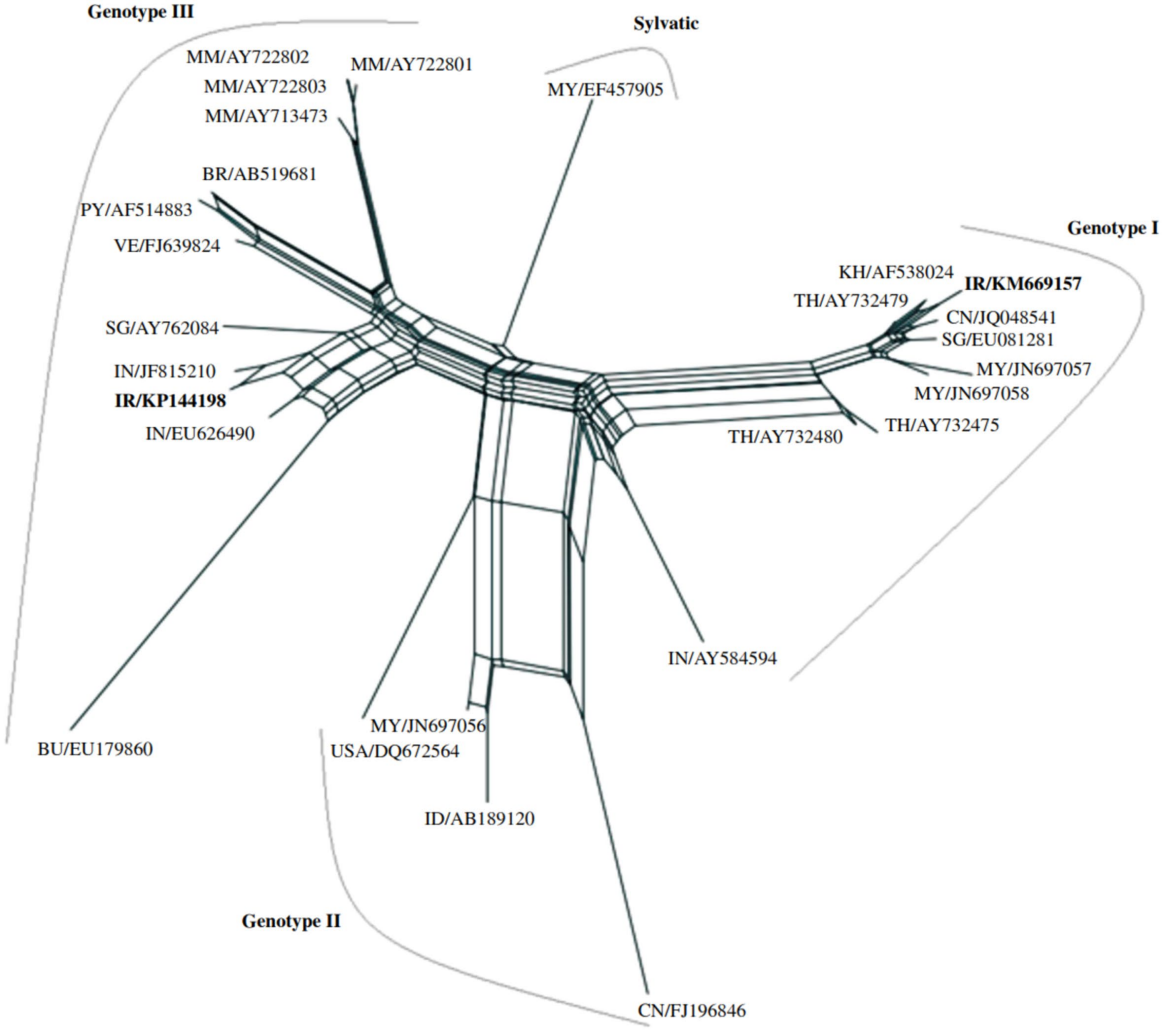
A neighbor-net network analysis was conducted to examine the phylogenetic relationships among DENV-1 genotypes, using partial nucleotide sequences of the capsid and premembrane genes (511 nucleotides) from various DENV strains. The standardized DENV strain nomenclature and corresponding GenBank accession numbers are provided for each sequence included in the analysis. DENV strains from Iran are highlighted in bold within the network (31).

Case studies demonstrate the importance of genomic surveillance in addressing DENV challenges. In one instance, a woman returning from India was diagnosed with DENV-2 Phylogenetic analysis showed her isolate was highly similar to Indian strains (12). In another case study, two patients with a travel history to Malaysia were found to carry DENV-1 genotype I and III strains (30). This finding underscores the significance of international travel in introducing new DENV strains to Iran, which could potentially result in locally transmitted cases. As such, continuous monitoring and surveillance of imported cases are essential for understanding their possible impact on the overall DENV burden in the country. Patient one’s sequence (KM669157) displayed a 72% similarity to a strain isolated from China (JQ048541). Upon further analysis, it was revealed that the patient’s strain clustered within genotype I, alongside other viral isolates from various Southeast Asian countries, including Malaysia, Cambodia, Singapore, Thailand, and China. This suggests a possible link between the spread of DENV-1 genotype I strains and travel within the region. On the other hand, patient two’s sequence (KP144198) showed a 52% similarity to a strain isolated from India (JF815210). This strain was found to form the third sub-lineage within genotype III, clustering with other isolates from Southeast Asian countries. Interestingly, genotype III exhibited three distinct sub-lineages, with all Myanmar isolates grouping together, indicating a close genetic relationship. Meanwhile, isolates from South America formed a separate sub-lineage, hinting at a possible divergence in the evolutionary history of DENV-1 genotype III strains in different geographical regions (31) (Figure 2). Due to the lack of phylogenetic analyses for other confirmed DENV cases, it cannot be concluded that the majority of DENV genotypes in Iran are the same as those reported in previous studies. Continuous monitoring of imported cases is essential for assessing their potential impact on the overall DENV burden in Iran. By implementing appropriate control measures, Iran can mitigate the risks associated with new DENV strains and ensure a robust public health response to DENV and other arboviral diseases.

## Seroprevalence, severe manifestations, and deaths caused by Dengue virus

While a comprehensive systematic review assessing the seroprevalence of DENV across Iran has yet to be conducted, several recent studies provide valuable insights into the prevalence of DENV antibodies in specific regions and populations. One large-scale study conducted from September 2017 to June 2018 collected 1,257 serum samples from six provinces: Bushehr, Hormozgan, Sistan and Baluchestan, Khuzestan (southern regions), and Gilan and Mazandaran (northern regions). The study included patients aged 15 years or older with a previous history of fever, headache, body ache, arthralgia, or rash illness. Using Euroimmune ELISA kits, 74 (5.9%) serum samples were found reactive for DENV IgG antibodies, with positive cases identified primarily in the southern regions of Khuzestan and Bushehr (32). In another study, investigators examined the seroprevalence of DENV in 60 suspected patients from southeastern Iran. The results showed that IgG antibodies were detected in 5% (3 patients) of the cohort (13). Although a nationwide systematic review of DENV seroprevalence is still needed to gain a complete understanding of the disease burden in Iran, these studies highlight the presence of DENV exposure in various regions and emphasize the importance of continued surveillance and targeted interventions. Further research is warranted to monitor the changing epidemiology of DENV in Iran and inform public health policies to mitigate the spread of the virus.

Dengue fever, as defined by the World Health Organization (WHO), is an acute febrile illness lasting 2–7 days, typically presenting with at least two of the following manifestations: headache, retro-orbital pain, myalgia, arthralgia, rash, hemorrhagic manifestation, and leukopenia (33). Iranian patients exhibit a range of severe manifestations that require careful monitoring and management. Leukopenia and thrombocytopenia have been identified as the most common laboratory abnormalities in Iranian patients with dengue fever (1, 14, 34). While these patients typically receive supportive care, including fluid resuscitation, electrolyte replacement, and transfusions when necessary, it is important to note that antiviral therapy is not routinely administered in these cases. Fortunately, despite the severity of symptoms, intensive care has not been required for most of the patients in question (13). The clinical course of dengue fever in Iranian patients is often characterized by high fever, with temperatures up to 38°C. Additional symptoms include headache, mild rash, bodily pain, nausea, lethargy, vomiting blood, and bone pain. Myalgia, in particular, has been reported with a severity score of 6–7 out of 10 in some cases (13, 14). The range of symptoms associated with DENV infections highlights the importance of prompt diagnosis and appropriate supportive care to prevent complications and ensure optimal patient outcomes.

DSS, a severe form of dengue characterized by hemorrhagic fever and circulatory failure, is associated with significantly high mortality rates. According to global estimates, approximately 390 million dengue infections and 96 million symptomatic cases occur annually, including over 2 million cases of DHF and around 15,000 deaths (35). The case-fatality rate of DHF is around 5%, with the World Health Organization estimating more than 20,000 deaths each year (36, 37). In Southeast Asia, the estimated lifetime number of dengue infections per person is around 3.3, with an overall annual infection rate of 5%. This rate is higher among children under 15 (12.5%) compared to adults (2.8%) (38). Although there is limited rigorous evidence on the mortality rate of DENV in Iran, sporadic deaths have been reported over the years, emphasizing the need for continued surveillance and research to better understand the disease’s burden and improve prevention strategies in the country.

## Dengue virus in Iran: a call for action

Addressing the risk of dengue epidemics in Iran is crucial, given the establishment of Ae. *aegypti* in southern Iran and the occurrence of dengue outbreaks in neighboring countries (18, 39). Despite the availability of dengue vaccines, their coverage in Iran remains extremely limited. This lack of vaccine deployment significantly hinders efforts to control the spread of dengue. In addition to vaccines, various vector control programs are being carried out in Iran, focusing on reducing mosquito populations through methods such as larval source management, insecticide spraying, and public health education campaigns. However, these measures face substantial limitations, including insecticide resistance, environmental concerns, and the high costs associated with long-term implementation. These limitations, coupled with the restricted vaccine coverage, continue to pose significant challenges in controlling dengue transmission in Iran. To effectively mitigate the disease burden and potential economic impact of DENV in Iran, implementing a comprehensive, strategic, and timely plan is essential. Several key steps can be taken to address this public health challenge: (1) Enhancing surveillance, diagnosis, and treatment: Strengthening molecular surveillance, prompt diagnosis, and appropriate treatment of DENV and other mosquito-borne diseases can help reduce transmission and improve patient outcomes. (2) Modeling data for monitoring and risk assessment: Utilizing genomic and serological data to model DENV evolution, spread, and the proportion of susceptible populations can help identify areas at risk for future epidemics. This approach should be supported by building local capacity and promoting a FAIR (Findable, Accessible, Interoperable, and Reusable) framework that emphasizes equity (28). (3) Improving understanding of transmission dynamics: Studying the spatiotemporal transmission dynamics of DENV at various geographical scales (municipality, province, and national levels) is crucial for refining mitigation strategies, including during inter-epidemic periods. (4) Implementing novel vector control approaches: The urgent need for new methods to control vectors and reduce transmission capacity must be addressed, as current strategies have been ineffective, costly, and environmentally problematic. (5) Developing and prioritizing vaccines: Efficient, affordable, and licensed vaccines for the most at-risk populations are vital to reduce DENV transmission and disease burden. While some promising vaccines have been licensed, prioritizing the most vulnerable groups will be essential to maximize the impact of future immunization programs (40). (6) Organizing training workshops and seminars, such as continuing medical education programs, can help change healthcare workers’ behavior and equip them with the knowledge and skills necessary to combat the spread of dengue effectively, as a recent study showed that the level of knowledge about dengue fever (DF) among healthcare providers is significantly inadequate (41, 42). By taking these proactive steps, Iran can minimize the risk of dengue epidemics and protect the well-being of its population.

## Conclusion

Addressing the risk of dengue epidemics in Iran is crucial, given the establishment of Ae. *aegypti* in southern Iran and dengue outbreaks in neighboring countries. Since no effective vaccine or specific treatment exists, health education and vector control are the most vital tools for prevention and control. Educating healthcare workers is particularly important, as they are responsible for disease prevention, control, and management. Investing in their capacity can improve dengue case management and the overall healthcare system.

## Data Availability

The original contributions presented in the study are included in the article/supplementary material, further inquiries can be directed to the corresponding author.
